# Correction: Glycyrrhetinic acid-modified redox-sensitive polymeric mixed micelles for tumor-specific intracellular delivery of cantharidin

**DOI:** 10.1039/d5ra90091c

**Published:** 2025-08-01

**Authors:** Yu Hu, Tian Lan, Ji Li, Lingjun Li, Jizheng Song

**Affiliations:** a School of Pharmacy, Shandong University of Traditional Chinese Medicine (TCM) 250355 Jinan Shandong China sdzyylilingjun@163.com songjizheng345@163.com; b Innovative Institute of Chinese Medicine, Shandong University of TCM 250355 Jinan Shandong China; c Affiliated Hospital of Shandong University of TCM 250011 Jinan Shandong China

## Abstract

Correction for ‘Glycyrrhetinic acid-modified redox-sensitive polymeric mixed micelles for tumor-specific intracellular delivery of cantharidin’ by Yu Hu *et al.*, *RSC Adv.*, 2024, **14**, 28753–28767, https://doi.org/10.1039/D4RA03171G.

Authors regret that an incorrect version of Fig. 6A was included in the originally published article. The correct version of Fig. 6 is shown herein.

**Fig. 1:**
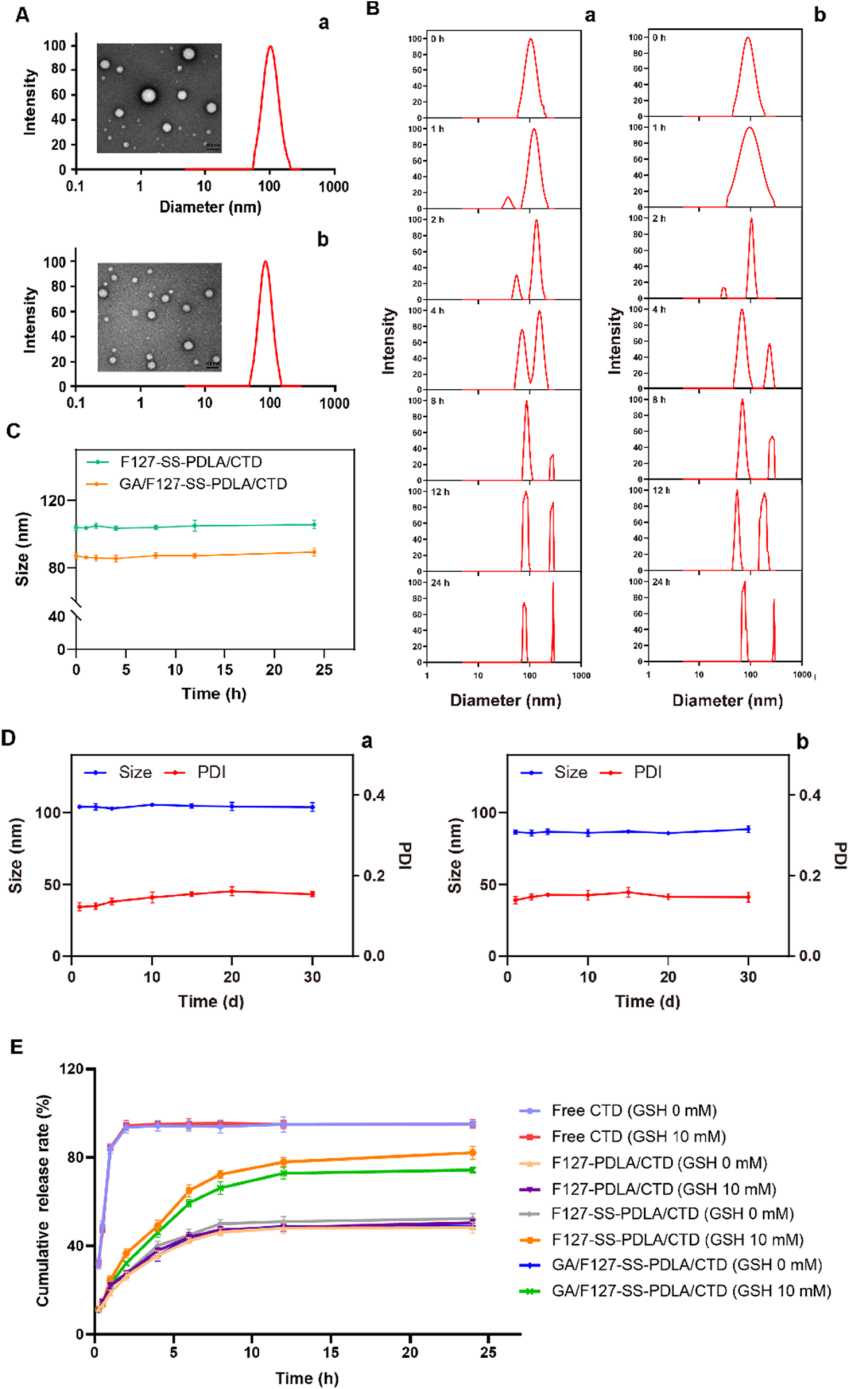
(A) Size distribution and morphology of F127-SS-PDLA/CTD (a) and GA/F127-SS-PDLA/CTD (b) micelles detected with DLS and TEM. The scale bar for TEM represents 100 nm. (B) Size distributions of F127-SS-PDLA/CTD (a) and GA/F127-SS-PDLA/CTD (b) micelles under reductive environment (10 mM GSH) for 24 h. (C) Changes in particle size of CTD-loaded micelles incubated with 10% FBS at 37 °C for 24 h. (D) Changes in particle size and PDI of F127-SS-PDLA/CTD (a) and GA/F127-SS-PDLA/CTD (b) micelles stored at 4 °C for 30 days. (E) Cumulative release profiles of free CTD and CTD in CTD-loaded micelles in normal saline with different GSH concentrations (0 and 10 mM). Data were presented as mean ± SD; *n* = 3.

The Royal Society of Chemistry apologises for these errors and any consequent inconvenience to authors and readers.

